# Deep learning in neurosurgery: a systematic literature review with a structured analysis of applications across subspecialties

**DOI:** 10.3389/fneur.2025.1532398

**Published:** 2025-04-16

**Authors:** Kivanc Yangi, Jinpyo Hong, Arianna S. Gholami, Thomas J. On, Alexander G. Reed, Pravarakhya Puppalla, Jiuxu Chen, Carlos E. Calderon Valero, Yuan Xu, Baoxin Li, Marco Santello, Michael T. Lawton, Mark C. Preul

**Affiliations:** ^1^The Loyal and Edith Davis Neurosurgical Research Laboratory, Barrow Neurological Institute, St. Joseph’s Hospital and Medical Center, Phoenix, AZ, United States; ^2^School of Computing and Augmented Intelligence, Arizona State University, Tempe, AZ, United States; ^3^School of Biological and Health Systems Engineering, Arizona State University, Tempe, AZ, United States

**Keywords:** artificial intelligence, convolutional neural network, deep learning, machine learning, neurological surgery, neurosurgery

## Abstract

**Objective:**

This study systematically reviewed deep learning (DL) applications in neurosurgical practice to provide a comprehensive understanding of DL in neurosurgery. The review process included a systematic overview of recent developments in DL technologies, an examination of the existing literature on their applications in neurosurgery, and insights into the future of neurosurgery. The study also summarized the most widely used DL algorithms, their specific applications in neurosurgical practice, their limitations, and future directions.

**Materials and methods:**

An advanced search using medical subject heading terms was conducted in Medline (via PubMed), Scopus, and Embase databases restricted to articles published in English. Two independent neurosurgically experienced reviewers screened selected articles.

**Results:**

A total of 456 articles were initially retrieved. After screening, 162 were found eligible and included in the study. Reference lists of all 162 articles were checked, and 19 additional articles were found eligible and included in the study. The 181 included articles were divided into 6 categories according to the subspecialties: general neurosurgery (*n* = 64), neuro-oncology (*n* = 49), functional neurosurgery (*n* = 32), vascular neurosurgery (*n* = 17), neurotrauma (*n* = 9), and spine and peripheral nerve (*n* = 10). The leading procedures in which DL algorithms were most commonly used were deep brain stimulation and subthalamic and thalamic nuclei localization (*n* = 24) in the functional neurosurgery group; segmentation, identification, classification, and diagnosis of brain tumors (*n* = 29) in the neuro-oncology group; and neuronavigation and image-guided neurosurgery (*n* = 13) in the general neurosurgery group. Apart from various video and image datasets, computed tomography, magnetic resonance imaging, and ultrasonography were the most frequently used datasets to train DL algorithms in all groups overall (*n* = 79). Although there were few studies involving DL applications in neurosurgery in 2016, research interest began to increase in 2019 and has continued to grow in the 2020s.

**Conclusion:**

DL algorithms can enhance neurosurgical practice by improving surgical workflows, real-time monitoring, diagnostic accuracy, outcome prediction, volumetric assessment, and neurosurgical education. However, their integration into neurosurgical practice involves challenges and limitations. Future studies should focus on refining DL models with a wide variety of datasets, developing effective implementation techniques, and assessing their affect on time and cost efficiency.

## 1 Introduction

Deep learning (DL), a subset of machine learning (ML), is an artificial intelligence (AI) method based on artificial neural networks that includes multiple layers of data processing to produce higher-level features. Artificial neural networks can combine various inputs to create a single input (1). Such technology holds substantial potential for improved pattern recognition and problem-solving in different medical disciplines, including neurosurgery. With the recent advancements in AI technologies, DL algorithms have begun to be integrated into neurosurgical practice in various ways.

For instance, DL technologies can improve surgical workflow analysis through real-time monitoring and video segmentation (2–7). DL can also potentially provide diagnostic support to surgeons by monitoring for adverse events or complications due to pathophysiological events during procedures (8). DL technologies may enhance the safety of neurosurgical procedures and provide a sense of reassurance to clinicians and patients by potentially diminishing intraoperative adverse events (9–11). On a related note, DL algorithms could allow for surgical instrument and motion tracking, allowing for more precise feedback intraoperatively and in teaching applications (12).

DL could also strengthen the ability to visualize and recognize complex anatomical structures by improving the accuracy of detection methods, including magnetic resonance imaging (MRI) and neuronavigation, and by identifying hemorrhages, spinal pathologies, and neuro-oncological conditions (13–17). Moreover, DL methods can be used to identify and classify intracranial lesions and perform volumetric assessments (18).

In this study, we elucidate prominent applications of DL algorithms in neurosurgery and provide evidence and examples of their current use within the field by conducting a systematic review of the existing literature. We also address future directions and limitations of these technologies. Because not all studies can be specifically expounded upon in such a review, we used representative articles to illustrate specific concepts and applications.

## 2 Materials and methods

A systematic search was conducted in PubMed, Embase, and Scopus databases on 8 November 2024 using the following keywords: (Neurosurgical Procedure) OR (neurosurgery) OR (neurologic surgery) OR (neurological surgery) OR (Procedure, Neurosurgical) OR (Procedures, Neurosurgical) OR (Surgical Procedures, Neurologic) OR (Neurologic Surgical Procedure) OR (Neurologic Surgical Procedures) OR (Procedure, Neurologic Surgical) OR (Procedures, Neurologic Surgical) OR (Surgical Procedure, Neurologic) AND ((Deep learning) OR (Learning, Deep) OR (Hierarchical Learning) OR (Learning, Hierarchical)).

These medical subject heading terms were linked with Boolean operators “AND” and “OR” to maximize the extent of coverage. An advanced search was conducted in PubMed using these medical subject heading terms. Then, the search was expanded by including Scopus and Embase database searches using the exact keywords. No time restrictions were applied. Our search words and articles were filtered by title or abstract. Duplications were excluded, and 2 independent neurosurgically experienced reviewers (A.G. and J.H.) screened the articles and examined all the full texts. A strict selection process was employed according to the Preferred Reporting Items for Systematic Reviews and Meta-Analysis (PRISMA) guidelines (19). Most publications included in the study were original research papers focused on DL applications in neurosurgical practice. Review articles, editorials, letters, and errata were excluded. Articles not published in English and articles for which the full text was unavailable were excluded. Studies that did not use DL algorithms and studies that used DL algorithms but did not apply them to neurosurgical practice were excluded. Finally, the reference lists of these articles were checked by 3 independent reviewers (J.H., A.S.G., K.Y.). Ultimately, all reviewers (J.H., A.S.G., K.Y., A.G.R., P.P.) agreed on the articles included in our study.

## 3 Results

**Figure 1:**
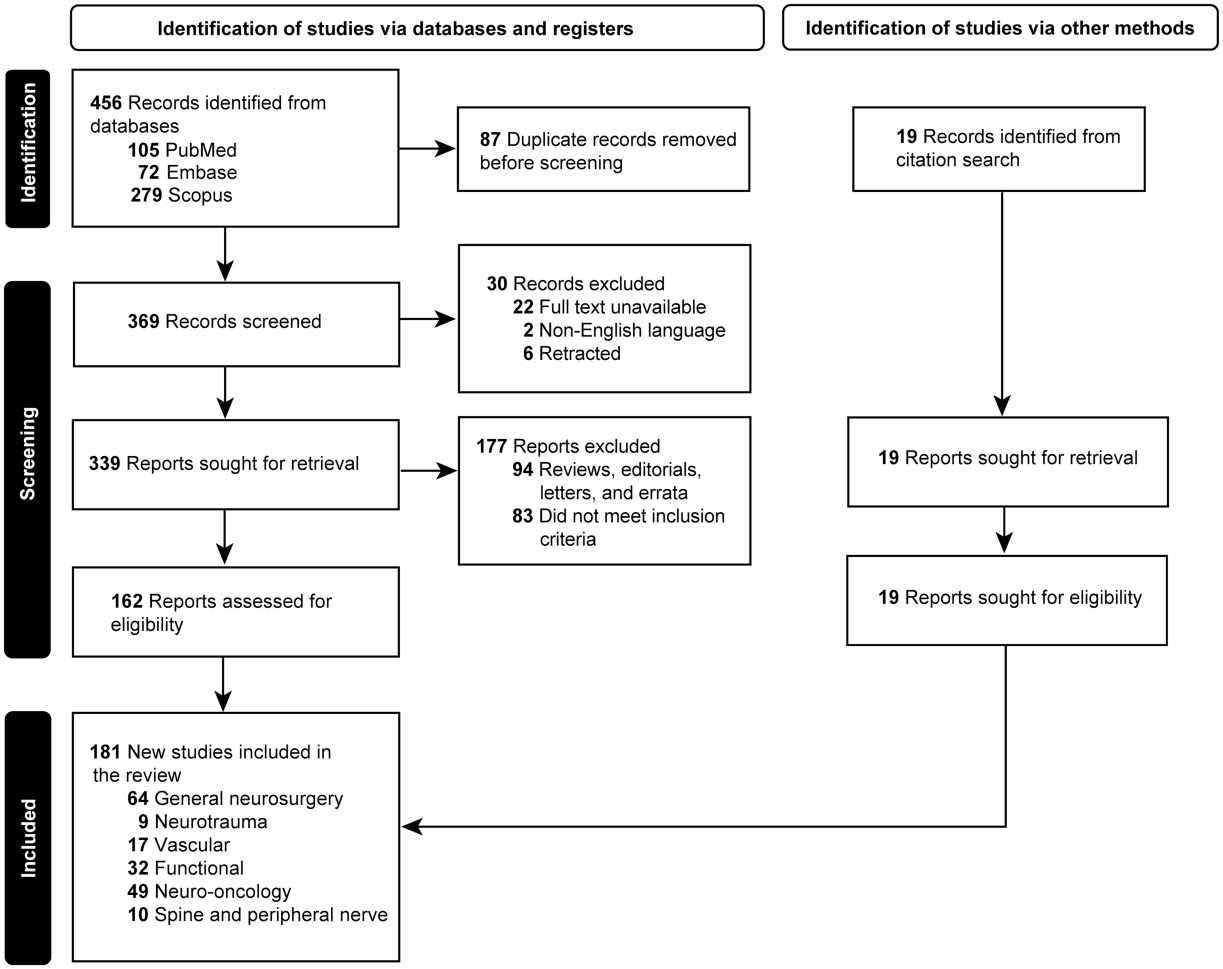
Flow diagram documenting the study selection process. *Used with permission from Barrow Neurological Institute, Phoenix, Arizona.*

Initially, 456 articles were retrieved. Eighty-seven duplicated papers were excluded. Twenty-two articles for which the full text was unavailable, 2 articles whose texts were unavailable in English, and 6 retracted articles were removed. Ninety-four articles determined to be letters, reviews, editorials, and errata were removed. Eighty-three articles that did not meet the inclusion criteria were excluded. The reference lists of the selected articles were screened by 3 independent reviewers (J.H., A.S.G., K.Y.), and 19 articles were found that were within the scope of our review and were included in the study. Upon completion of the screening, 181 articles were found eligible for the study and included (2, 3, 7, 9–11, 13–18, 20–188). The selection process is depicted in the PRISMA flowchart in Figure 1.

Included articles were divided into 6 groups according to the subspecialty: spinal surgery and peripheral nerve (*n* = 10) (Table 1) (14, 20–26, 168, 182), neurotrauma (*n* = 9) (Table 2) (27–35), vascular neurosurgery (*n* = 17) (Table 3) (9, 10, 13, 36–45, 166, 169, 179, 186), functional neurosurgery (*n* = 32) (Table 4) (46–76, 187), neuro-oncology (*n* = 49) (Table 5) (2, 3, 7, 15, 16, 18, 77–114, 175–178, 188), and general neurosurgery (*n* = 64) (Table 6) (11, 17, 115–165, 167, 170–174, 180, 181, 183–185).

**Table 1:** Studies that applied deep learning algorithms in spinal and peripheral nerve surgery.

Study (year)	Data used	Procedure or goal
Yu et al. (2020) (20)	CT and 3D reconstructed images	Percutaneous endoscopic spinal surgery
Zhong et al. (2020) (26)	Peripheral nerve micro-CT images	Obtaining contours of fascicular groups from micro-CT images of the peripheral nerve
Agaronnik et al. (2022) (22)	Neuromonitoring documentation	Intraoperative neuromonitoring in spine surgery
Kim et al. (2022) (24)	Radiographic datasets	Spinal surgery
Bakaev et al. (2023) (23)	Radiographic datasets	Spinal imaging
Ghauri et al. (2023) (14)	Kaggle dataset of 967 spinal radiographs	Classification of certain degenerative spinal conditions
Massalimova et al. (2023) (21)	Multiple vibroacoustic sensors, such as a contact microphone, a free-field microphone, a tri-axial accelerometer, a uni-axial accelerometer, and an optical tracking system	Spinal pedicle drilling
Jawed et al. (2024) (168)	Surgical video datasets	Microdiscectomy surgical video annotation
Martino Cinnera et al. (2024) (25)	Demographics and clinical characteristics	Lumbar stenosis
Mehandzhiyski et al. (2024) (182)	Radiographic datasets and common medical information from patients with single-level herniation	Creation of a predictive score for lumbar disc reherniation after microdiscectomy without fusion

3D, 3-dimensional; CT, computed tomography.

**Table 2:** Studies that applied deep learning algorithms in neurotrauma.

Study (year)	Data used	Procedure or goal
Matzkin et al. (2020) (35)	Postoperative CT	Decompressive craniectomy after TBI
Adil et al. (2022) (30)	TBI patient data	Prediction of TBI outcomes in the low-resource setting
Koschmieder et al. (2022) (34)	Magnetic resonance imaging datasets	Detection of cerebral microbleeds in TBI patients
Agrawal et al. (2023) (29)	Head CT	Automated intracranial hemorrhage detection in TBI
Biswas et al. (2023) (31)	Patient referral data	Predicting chronic subdural hematoma referral outcomes
Gençtürk et al. (2023) (33)	CQ500 dataset	Subdural hemorrhage
Jiang et al. (2023) (28)	Noncontrast CT	TBI detection
Agrawal et al. (2024) (27)	CT datasets	Midline shift detection in TBI
Vargas et al. (2024) (32)	CT datasets	Chronic subdural hematoma

CT, computed tomography; TBI, traumatic brain injury.

**Table 3:** Studies that applied deep learning algorithms in vascular neurosurgery.

Study (year)	Data used	Procedure or goal
Hoffmann et al. (2016) (44)	Postoperative CT	Intraoperative analysis of cortical perfusions
Danilov et al. (2020) (40)	CT datasets	Classification of intracranial hemorrhage subtypes
Nishi et al. (2021) (39)	Noncontrast CT	CT diagnosis of nontraumatic SAH by nonspecialists
Pangal et al. (2021) (169)	Surgical video datasets	Prediction of blood loss and vascular injury control of the ICA
Voter et al. (2021) (38)	Head CT	Detection of intracranial hemorrhage
Kugener (2022) (10)	Image and video datasets	Detection of blood loss and aid in bleeding control in ICA hemorrhage
Su et al. (2022) (9)	MR CLEAN registry, MR CLEAN-NOIV trial, and the HERMES collaboration datasets	To detect intracranial vessel perforation in DSA during endovascular thrombectomy
Venugopal et al. (2022) (41)	Real-time intraoperative blood vessel segmentation of brain vasculature with a 3D reconstruction algorithm and virtual-fixture-based strategy to control Micron	To prevent intraoperative vascular damage
Wang et al. (2022) (36)	CTA dataset	Diagnosis of cerebral aneurysm
Wang et al. (2022) (43)	Sections specimens	Diagnosis of cerebral cavernous malformations
Angkurawaranon et al. (2023) (37)	CT datasets	Detection and classification of intracerebral hemorrhage
Balu et al. (2023) (45)	Video dataset	Surgeon performance assessment in a cadaveric carotid artery injury control simulation
Tong et al. (2023) (13)	CT	Intraparenchymal and intraventricular hemorrhage for catheter puncture path planning
Xu et al. (2023) (42)	DSA sequences	Classification of stages of moyamoya disease
García-García et al. (2024) (166)	Noncontrast CT	Blood segmentation for spontaneous aneurysmal SAH
Kiewitz et al. (2024) (186)	Cranial CT	Automated multiclass segmentation of structures and pathologies relevant for aneurysmal SAH outcome prediction
Won et al. (2024) (179)	MRI dataset (susceptibility-weighted imaging)	Detection and anatomical localization of cerebral microbleeds

CT, computed tomography; CTA, computed tomography angiography; DSA, digital subtraction angiography; HERMES, Highly Effective Reperfusion Evaluated in Multiple Endovascular Stroke Trials; ICA, internal carotid artery; MR CLEAN, Multicenter Randomized Clinical Trial of Endovascular Treatment for Acute Ischemic stroke in the Netherlands; MRI, magnetic resonance imaging; SAH, subarachnoid hemorrhage.

**Table 4:** Studies that applied deep learning algorithms in functional neurosurgery.

Study (year)	Data used	Procedure or goal
Souriau et al. (2018) (66)	Data collected from 8 intracortical channels from different cortical regions	DBS
Bermudez et al. (2019) (64)	Simulation coordinates with 3D patches extracted and associated with an efficacy score	DBS
Onofrey et al. (2019) (70)	Clinical epilepsy imaging data	Segmentation of the brain surface in postsurgical CT of epilepsy patients following surgical implantation of electrodes
Yokota et al. (2019) (75)	T1 and T2 MRI	Real-time estimation of electric fields induced by transcranial magnetic stimulation
Baxter et al. (2020) (63)	T1- and T2-weighted MRI datasets	Localization of subthalamic nucleus in MRI
Liu et al. (2020) (62)	Whole-brain MRI and cropped volume	Anterior thalamus targeting for DBS
Martineau et al. (2020) (76)	Local field potentials	Action decoding based on subthalamic local field potentials
Peralta et al. (2020) (65)	Microelectrode recordings	Localization of subthalamic nucleus
Cui et al. (2021) (61)	Contrast-enhanced T1-weighted MRI	DBS
Edwards et al. (2021) (74)	MRI	Automated landmark localization for neuronavigation
Jiang et al. (2021) (60)	Video interviews	Classification of major depressive disorder and response to DBS
Uneri et al. (2021) (73)	Fluoroscopy images	Neuroelectrode placement
Baker et al. (2022) (54)	Intraoperative kinematic recordings	DBS
Baxter et al. (2022) (56)	T1- and T2-weighted MRI datasets	Localization of subthalamic nucleus in MRI
Chen et al. (2022) (59)	Preoperative images and intraoperative sparse data	Estimating shift at brain surface in DBS
Gao et al. (2022) (57)	Historical data	DBS
Hosny et al. (2022) (55)	Local field potentials	DBS
Liu et al. (2022) (58)	MRI datasets	DBS
Zhang et al. (2022) (53)	MRI datasets	DBS
Chen et al. (2023) (48)	Prior-enhanced multiobject MRI segmentation	DBS
Chen et al. (2023) (51)	Finite-element biomechanical brain model	DBS
Joseph et al. (2023) (50)	MRI datasets	DBS
Makaram et al. (2023) (68)	Interictal intracranial EEG data	Epileptogenic zone and guided neurosurgery
Rui-Qiang et al. (2023) (52)	MRI datasets	DBS
Zheng et al. (2023) (49)	Anatomical information from publicly available high-quality datasets	DBS
Al-Jaberi et al. (2024) (46)	CT datasets	Applicability of image fusion of highly resolved flat detector CT to CT for electrode placement in DBS
Caredda et al. (2024) (71)	Segmentation of brain cortex	Functional brain mapping
Courtney et al. (2024) (69)	MRI datasets	Imaging in epilepsy surgery
Eid et al. (2024) (47)	EEG datasets	DBS
Ho et al. (2024) (187)	Cranial MRI	Segmentation of the subthalamic nucleus on clinical MRI thus minimize variability in subthalamic nucleus targeting and eliminate human biases
Li et al. (2024) (72)	Preoperative MRI	Image-guided open cranial surgery
Maged et al. (2024) (67)	Image datasets	Subthalamic nucleus localization in DBS

CT, computed tomography; DBS, deep brain stimulation; EEG, electroencephalography; MRI, magnetic resonance imaging.

**Table 5:** Studies that applied deep learning algorithms in neuro-oncology.

Study (year)	Data used	Procedure or goal
Izadyyazdanabadi et al. (2017) (176)	Image datasets	Discrimination of diagnostic CLE images among thousands of nondiagnostic images to support the diagnosis of brain tumors
Izadyyazdanabadi et al. (2018) (177)	Image datasets	Creation of a CNN model for the automatic selection of diagnostic CLE images to aid in the rapid diagnosis of brain tumors
Li et al. (2018) (109)	Probe-based CLE data	Context-aware decision support in neurosurgical oncology based on an efficient classification of endomicroscopic data
Chang et al. (2019) (18)	Preoperative and postoperative MRI datasets	Evaluation of glioma burden (fully automated volumetric measurement)
Fabelo et al. (2019) (79)	Hyperspectral images of in vivo human brain tissue	In vivo identification of glioblastoma tumor using hyperspectral images of the human brain
Izadyyazdanabadi et al. (2019) (175)	Image datasets	Improvement in the quality of CLE images to support the diagnosis of brain tumors
Carton et al. (2020) (78)	Intraoperative ultrasound images	Automatic segmentation of brain tumor in intraoperative ultrasound images
Colecchia et al. (2020) (111)	Clinical data	Brain tumor segmentation
Ermiş et al. (2020) (110)	MRI datasets	Fully automated brain resection cavity delineation for radiation target volume definition in glioblastoma
Franco et al. (2020) (112)	MRI and magnetic resonance spectroscopy	Spectroscopic prediction of brain tumors
Rahmat et al. (2020) (92)	Multiple MRI sequences	Multiscale segmentation in glioblastoma treatment
Chen et al. (2021) (108)	MRI datasets (contrast-enhanced images of the 3-dimensional MPRAGE sequences)	Automatic meningioma segmentation and grading prediction
Di Ieva et al. (2021) (93)	Brain tumor images, histological specimens, connectomics data	Segmenting brain tumors, predicting genetic subtypes, and extent of resection
Lee et al. (2021) (91)	MRI datasets	Imaging analysis of vestibular schwannoma after radiosurgery
Shen et al. (2021) (106)	Tissue samples and fluorescence images	Real-time intraoperative glioma diagnosis
Wu et al. (2021) (105)	Stereotactic radiosurgery dataset	Segmentation of various brain lesions for radiosurgery
Zeineldin et al. (2021) (107)	Public dataset of high-grade glioma patients	Brain tumor segmentation
Danilov et al. (2022) (89)	Preoperative MRI	Subtyping gliomas
Danilov et al. (2022) (90)	MRI datasets	MRI-guided typing of brain gliomas
Fang et al. (2022) (88)	Fine-grained texts of clinical records	Extraction of clinical named entity for pituitary adenomas
Khan et al. (2022) (2)	Surgical video datasets	Performance of surgical workflow analysis of endoscopic pituitary adenoma surgery
Madhogarhia et al. (2022) (77)	Multiparametric MRI	Pediatric brain tumor automatic segmentation
Reinecke et al. (2022) (15)	Stimulated Raman histology images of intraoperative tissue samples	Intraoperative tumor detection
Wu et al. (2022) (87)	Intraoperative images	Resolution enhancement and classification of tumors
Zeineldin et al. (2022) (104)	Interventional ultrasound data	Brain tumor automatic segmentation
Fischer et al. (2023) (7)	Surgical video datasets	Annotation of endoscopic pituitary tumor removal surgical videos
Kang et al. (2023) (178)	MRI	MRI segmentation and volumetric assessment of intracranial meningiomas
Li et al. (2023) (99)	Motion of patient skull and surgical drill from stereo microscopic videos	Anatomy and skull base surgery
Luckett et al. (2023) (83)	Multimodal imaging	Glioblastoma survival prediction
Pirhadi et al. (2023) (100)	Image datasets	Ultrasound-guided brain tumor resection
Prathaban et al. (2023) (97)	Brain tissue histology	Detection of tumor infiltration in diffuse gliomas
Puustinen et al. (2023) (84)	Hyperspectral imaging	Brain tumor surgery
Salari et al. (2023) (101)	RESECT dataset	MRI intraoperative ultrasound in brain tumor surgery
Salari et al. (2023) (102)	RESECT dataset	Multimodal anatomical landmark detection; MRI intraoperative ultrasound in brain tumor surgery
Srikanthan et al. (2023) (103)	Tissue burns	Glioblastoma detection using mass spectrometry
Touati and Kadouri (2023) (113)	Brain tumor dataset (BraTS2018)	Generation of reliable MRI contrasts with enhanced tumors
Wang and Ye (2023) (114)	Datasets of BraTS2018 and BraTS2019	Multimodality glioma MRI
Wang et al. (2023) (85)	MRI datasets	Short-term postoperative facial nerve function in acoustic neuroma patients
Zanier et al. (2023) (86)	MRI datasets	Volumetric assessment of variable grade gliomas
Zhu et al. (2023) (98)	SEER database	Surgical options for patients with low-grade glioma
Bianconi et al. (2023) (188)	Pre-and postoperative MRI datasets	To train an algorithm for GBM segmentation on a clinical MRI dataset and to obtain reliable results
Castiglioni et al. (2024) (80)	MRI datasets	Pediatric craniopharyngioma diagnosis
Cekic et al. (2024) (96)	MRI and surgical microscope images	Brain tumor surgery
Da Mutten et al. (2024) (82)	MRI datasets	Pituitary adenoma volumetric assessment
Hsu et al. (2024) (95)	Ex vivo specimens	Brain tumor grading
Khan et al. (2024) (3)	Surgical video datasets	Artificial intelligence–assisted coaching for endoscopic pituitary adenoma surgery
Wang et al. (2024) (94)	Image datasets	Differentiation of central nervous system tumors
Zeineldin et al. (2024) (16)	Neuronavigation	Brain tumor segmentation, patient registration, and explainable output prediction in brain tumor surgery
Zhu et al. (2024) (81)	SEER 18 database	Glioblastoma survival prediction and surgery recommendation

BraTS, Brain Tumor Segmentation; CLE, confocal laser endomicroscopy; CNN, convolutional neural network; GBM, glioblastoma multiforme; MPRAGE, magnetization-prepared rapid gradient-echo; MRI, magnetic resonance imaging; RESECT, Retrospective Evaluation of Cerebral Tumors; SEER, Surveillance, Epidemiology, and End Results.

**Table 6:** Studies that applied deep learning algorithms in general neurosurgery.

Study (year)	Data used	Procedure or goal
Moccia et al. (2018) (139)	Microscopy images	Improvement of safety in neurosurgery with an active handheld instrument
Danilov et al. (2019) (138)	Operative reports	Postoperative hospital stay prediction in neurosurgery
Nitsch et al. (2019) (142)	Intraoperative USG images	Image-guided neurosurgery
Shi et al. (2019) (141)	MRI	Automatic segmentation of brainstem
Canalini et al. (2020) (134)	Resection cavities in USG volumes	Comparison of USG images obtained at different phases of the tumor resection
Danilov et al. (2020) (136)	Word-embedded reports of primary surgical cases	Prediction of postoperative hospital stay in neurosurgery based on operative reports
Drakopoulos et al. (2020) (137)	Preoperative and intraoperative MRI	Neuronavigation
Farnia et al. (2020) (135)	Generated synthetic database of vessels	Intraoperative photoacoustic imaging
Li et al. (2020) (143)	Complete skulls from the public head CT collection CQ500	Skull defect restoration and cranial implant generation for cranioplasty
Lucena et al. (2020) (145)	Multishell diffusion MRI and paired synthetic single-shell diffusion MRI	MRI tractography to help locate critical white matter tracts
Matzkin et al. (2020) (144)	CT and simulated virtual craniectomy	Cranial implant design via virtual craniectomy
Davids et al. (2021) (131)	Surgical videos	Microsurgery
Han et al. (2021) (133)	MRI and CT	Representation of surgical tool motion and objective assessment of microsurgical skills
Li et al. (2021) (150)	High-resolution healthy skulls	Skull defect restoration and cranial implant generation for cranioplasty
Mahapatra et al. (2021) (146)	Recorded USG images	Automatic detection of cotton balls during brain surgery
Pangal et al. (2021) (167)	Video datasets	Annotation of neurosurgical intraoperative videos
Quon et al. (2021) (149)	Manual ventricle segmentation and volume calculation values	Automatic cerebral ventricle segmentation and volume calculation
Ramesh et al. (2021) (132)	Intraoperative neurosurgical videos	Tool detection and characterization
Staartjes et al. (2021) (170)	Image datasets	Identification of anatomic structures during endoscopic endonasal approach
Zeineldin et al. (2021) (147)	MRI and interventional USG images	Brain shift estimation
Zeineldin et al. (2021) (148)	Preoperative MRI and interventional USG images	Brain shift compensation
Abramson et al. (2022) (125)	USG datasets	Foreign body objects detection in neurosurgical procedures
Feng et al. (2022) (157)	Image datasets	Patient head pose estimation
Gaur et al. (2022) (152)	Preoperative MRI volumes	Image-guided neurosurgery
Han et al. (2022) (126)	MRI and CT	Minimally invasive intracranial neurosurgery
Han et al. (2022) (127)	Simulated dataset (simulated cone-beam CT and simulated deformations)	MRI cone-beam CT image registration for neurosurgical guidance
Korycinski et al. (2022) (154)	MRI	Neural fiber prediction
Lam et al. (2022) (130)	Individual admissions data	Neurosurgery inpatient outcome prediction for discharge planning
Li et al. (2022) (155)	2D image scene dataset, 3D point cloud scene	2D and 3D working scene in neurosurgery
McKinley et al. (2022) (151)	Mueller polarimetric images of fixed human brain sections	White matter fiber tract visualization
Pangal et al. (2022) (11)	Surgical videos	Prediction of outcome of surgical hemorrhage
Philipp et al. (2022) (156)	Image datasets	Surgical instrument activity in neurosurgery
Su et al. (2022) (128)	RGB-D camera	Image-guided neurosurgery
Yilmaz et al. (2022) (124)	Neurosurgeon and student performance data in 156 virtually simulated tumor resection tasks	Simulated tumor resection task
Zhang et al. (2022) (153)	Cone-beam CT	Image-guided neurosurgery
Zufiria et al. (2022) (129)	Interventional MRI	MRI-guided neurosurgery
Baghdadi et al. (2023) (118)	Sensorized bipolar forceps, SmartForceps System use data	Neurosurgical performance analysis
Chiou et al. (2023) (121)	Augmented reality surgical navigation system	Surgical navigation
Danilov et al. (2023) (120)	Neurosurgical operative reports	Classification of neurosurgical operative reports
Eskandari et al. (2023) (159)	Preoperative T1-weighted MRI volumes, preresection intraoperative USG volumes of the BITE database	Image-guided neurosurgery
Gonzalez-Romo et al. (2023) (174)	Video datasets	Identification of gross and fine hand movements during microvascular anastomosis simulation
Haber et al. (2023) (161)	Head CT	Detection of idiopathic normal pressure hydrocephalus
Sarwin et al. (2023) (158)	Video datasets	Minimally invasive neurosurgery navigation
Shimamoto et al. (2023) (123)	Preoperative and intraoperative MRI	Brain shift prediction and neurosurgical navigation
Xu et al. (2023) (119)	Force data obtained from a novel sensorized surgical glove	Microsurgery
Yoon et al. (2023) (160)	Noncontrast head CT	Interpretation of urgent head CT
Zhang et al. (2023) (122)	Intraoperative cone-beam CT	Image-guided neurosurgery
Bi et al. (2024) (165)	MRI	Neuronavigation
Bobeff et al. (2024) (185)	Cranial CT	Automation of segmentation of intracranial compartments and analysis of cerebrospinal fluid distribution
de Boer et al. (2024) (162)	MRI	Brain, skin, tumor, and ventricle segmentation
Matasyoh et al. (2024) (164)	Neuroendoscopic images	Facilitating authentic and interactive learning experiences in neuroendoscopy
Moriconi et al. (2024) (163)	Polarization states intensity images	Provision of visual feedback on white matter fiber bundle orientation
On et al. (2024) (172)	Video datasets	Measurement of surgical motion during a cadaveric mastoidectomy procedure
On et al. (2024) (173)	Video datasets	Detection of hand motion during microvascular anastomosis simulation
Park et al. (2024) (17)	Image datasets	Improvement of the quality of images collected from exoscopes and surgical microscopes (sharpness, distortion) and measurement of the depth of the surgical field
Payman et al. (2024) (180)	Image datasets	Identification of skull-base foramina to enhance anatomical education and intraoperative structure visualization
Rahmani et al. (2024) (116)	MRI datasets	Neuronavigation
Rhomberg et al. (2024) (115)	Skull radiographs or scout CT	Proper identification of shunt valves on radiographs
Sastry et al. (2024) (117)	MRI and USG datasets	Neurosurgical inpatient admissions
Sugiyama et al. (2024) (171)	Video datasets	Video analysis of instrument motion in microvascular anastomosis training
Tan et al. (2024) (140)	Preoperative paired CT and contrast-enhanced MRI	Generation of synthetic high-resolution CT from MRI through a conditional generative adversarial network
Wodzinski et al. (2024) (181)	Large-scale 3D volumetric data	Easier and faster modeling of cranial implants
Zanier et al. (2024) (183)	Surgical video datasets	Enhancement of intraoperative orientation
Zanier et al. (2024) (184)	Cranial CT	Exploration of the feasibility of generating synthetic CT imaging from radiographs

2D, 2-dimensional; 3D, 3-dimensional; BITE, Brain Images of Tumors for Evaluation; CT, computed tomography; MRI, magnetic resonance imaging; RGB-D, red-green-blue depth; USG, ultrasonography.

**Figure 2:**
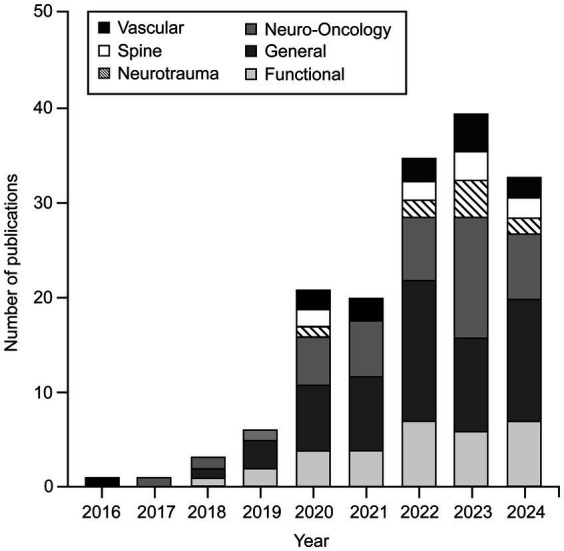
Number of publications focused on applying various deep learning (DL) algorithms in neurosurgical procedures by year and subspecialty. This bar chart shows that applications of DL algorithms in neurosurgery were little studied in 2016. Such studies began to gain popularity in 2019, and the trend has continued to grow. By November 2024, the publication numbers had nearly matched those for 2023. Although these algorithms have been applied across almost all neurosurgery subspecialties, general neurosurgery, neuro-oncology, and functional neurosurgery are the leading subspecialties in using DL algorithms. *Used with permission from Barrow Neurological Institute, Phoenix, Arizona.*

Although DL applications have been widely used in different subspecialties of neurosurgery, our findings indicate that there are leading subspecialties that are at the forefront of this technological advancement. General neurosurgery (*n* = 64), functional neurosurgery (*n* = 32), and neuro-oncology (*n* = 49) are the leading subspecialties, as shown in Figure 2.

The procedures that most commonly used DL algorithms were deep brain stimulation (DBS) and subthalamic and anterior thalamic nuclei localization (*n* = 24) in the functional neurosurgery group (Table 4); segmentation, identification, classification, and diagnosis of various brain tumors (*n* = 29) in the neuro-oncology group (Table 5), and neuronavigation and image-guided neurosurgery (*n* = 13) in general neurosurgery group (Table 6). Computerized tomography (CT), MRI, and ultrasonography images (*n* = 79) were found to be the most widely used datasets in all groups to train DL architectures, followed by surgical videos and various types of image datasets, including radiography, digital subtraction angiography, surgical microscopy, and neuroendoscopy images (*n* = 51).

To the best of our knowledge, interest in applying DL algorithms in neurosurgery began in the 2010s, when the first studies were published. Although very few articles were published on this topic in 2016, more studies were published beginning in 2019, and this trend has continued to grow. The publication numbers during 2024 nearly matched those for 2023 (Figure 2).

## 4 Discussion

### 4.1 DL

ML is an AI method that enables computers to process data and learn valuable patterns, facilitating better user decision-making. DL is a subset of ML that focuses on mimicking the complex neuron structures found in the human brain by using multiple layers of latent units to compose a neural network. It is widely recognized as an effective tool for data analysis due to its ability to understand the intricate nature of data.

There are various types of DL neural network models designed to simulate different real-world scenarios: multilayer perceptron, convolutional neural network (CNN) (189), recurrent neural network (RNN) (190), graph neural network (191), generative adversarial network (192), transformer (193), and diffusion model (194, 195), among others. Many variant neural network models, such as long short-term memory (196) and denoising diffusion probabilistic model (195), have been developed to address specific limitations and meet the challenge of significantly changing real-world use cases. Some variations, such as the vision transformer, are designed as fusions of different neural network modules to leverage their superior performance (197). Motivated by this flexibility in neural network architecture design, researchers can better facilitate their specific purposes.

In recent years, with the continuing advancements in the fields of AI and medicine, various architectural designs of DL have started to be used in neurosurgery (Table 7) (37, 38, 41, 73, 74, 81–83, 88, 115, 116, 123, 125, 137). This study focuses on the applications of the most widely used deep neural architecture designs within the neurosurgical practice.

**Table 7:** Brief description of the most widely used neural network models in neurosurgical studies.

Technique	Description	Example studies
Multilayer perceptron	A feedforward artificial neural network consisting of multiple layers of fully connected neurons; it has a well-recognized capability of distinguishing data that is not linearly separable	(81, 83, 137)
Convolutional neural network	A regularized feed-forward neural network based on the shared-weight architecture of the convolution kernels (filters); widely used to learn the spatial relationship (or visual patterns) among adjacent pixels within an image	(37, 38, 41, 73, 74, 82, 115, 123, 125)
Long short-term memory	An advanced recurrent neural network is composed of a cell, an input gate, an output gate, and a forget gate; its insensitivity to gap length gives it an advantage over conventional recurrent neural network models in modeling the temporal transitions of sequential data	(88)
Generative adversarial network	A prominent framework for approaching generative artificial intelligence consists of 2 neural networks that contest each other, where the generator is trained to fool the discriminator, and the discriminator is trained to reject the poor output	(116)

### 4.2 DL applications in neurosurgical practice

Over the past several years, the development of AI, specifically DL techniques, has shown considerable potential in neurosurgery (198). Various DL architectural designs are used in neurosurgical practice for purposes that include operative video analysis, predicting outcomes, microsurgical skill assessments, diagnostic support, volumetric assessment, and neurosurgical education. This section will examine the most commonly used applications of DL in neurosurgical training.

#### 4.2.1 Operative video and image analysis

DL CNNs can analyze surgical videos through computer vision (CV) (199). This approach analyzes operative video to define surgical workflows by establishing start and end points for various stages of a procedure and annotating clinically relevant details, such as anatomical landmarks and instrument detection, aiding in assessment, real-time monitoring, and surgical coaching (2–6).

Many CV pipelines share common elements, beginning with creating a dataset composed of individual surgical images (frames) annotated with overlays that highlight tools, anatomical structures, or the stage of the operation. These data are often generated manually and require expert evaluation. CV models are trained on this ground-truth data and tested on new images or videos. The successful implementation of CV models relies heavily on the quality, quantity, and accuracy of these annotated video sets (167).

Neurosurgeons often use exoscopes, microscope-exoscope hybrid systems, and endoscopes, all equipped with cameras to capture surgical video. Neurosurgical procedures are frequently recorded, resulting in surgeons accumulating hundreds of hours of surgical video. Surgeons use this video for educational purposes, cropping it to highlight important parts of the surgery and discuss critical aspects. To date, CV has primarily been applied to neurosurgery with 2 main focuses: workflow analysis and video segmentation.

**Figure 3:**
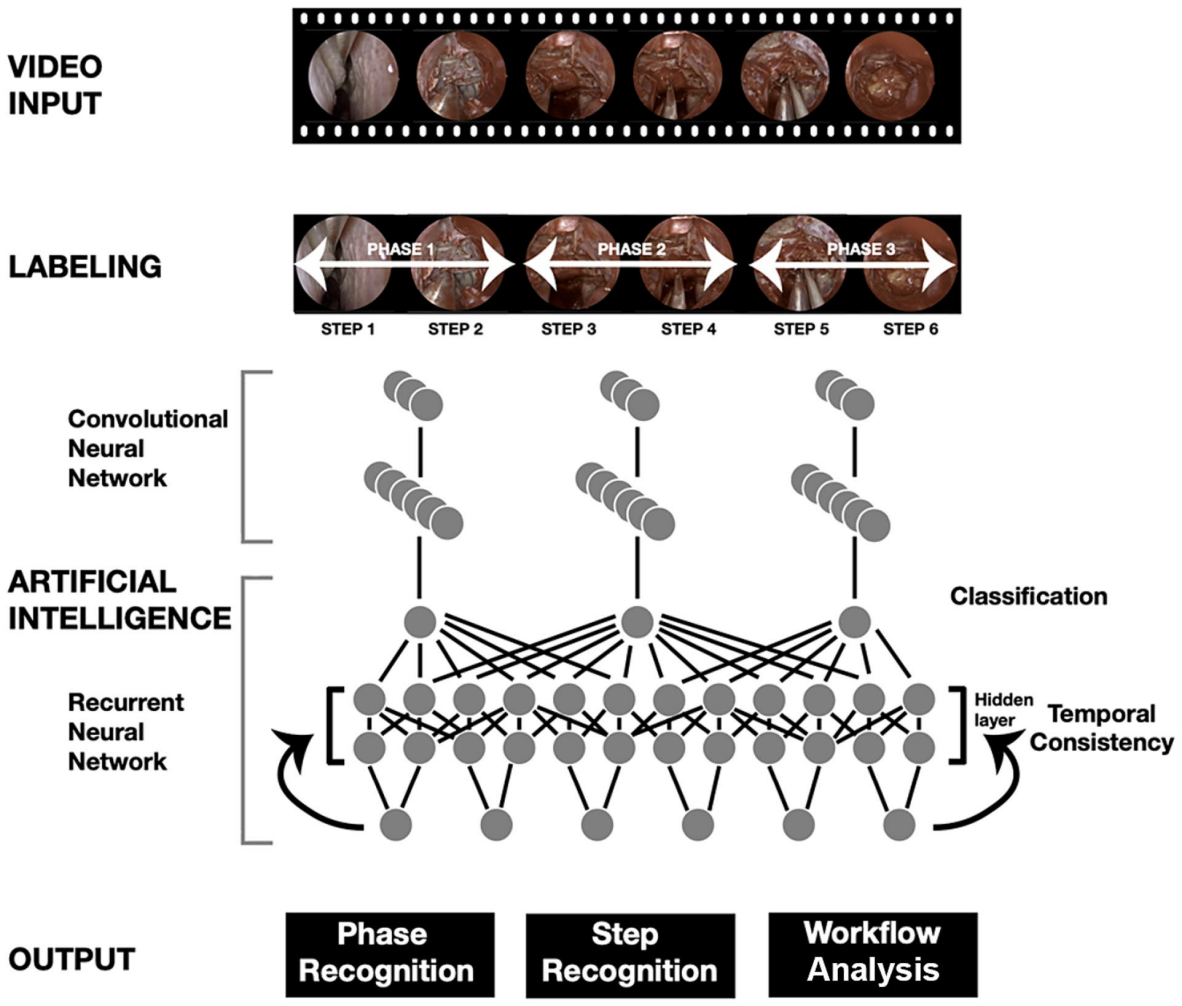
Use of deep learning (DL) algorithms in surgical workflow analysis. The workflow diagram adapted from Khan et al. (2) illustrates the phase and step recognition process in surgical videos using artificial intelligence. The process begins with a video input that undergoes labeling into phases and steps. A convolutional neural network (CNN) extracts features from the labeled video frames, whereas a recurrent neural network (RNN) ensures temporal consistency across the video sequence. The combined CNN and RNN architecture enables accurate classification of surgical phases, recognition of specific steps, and comprehensive workflow analysis. The final output provides detailed insight into phase recognition, step recognition, and overall surgical workflow*. Used with permission from Khan DZ, Luengo I, Barbarisi S, et al. Automated operative workflow analysis of endoscopic pituitary surgery using machine learning: development and preclinical evaluation (IDEAL stage 0). J Neurosurg. 2022;137* (1)*:51–58. doi: 10.3171/2021.6.JNS21923.*

Operative workflow analysis deconstructs operations into distinct steps and phases. Each operative video is labeled with timestamps corresponding to these steps and stages. These labeled data and the video are fed into DL models, enabling automatic recognition and analysis of these components. This process allows for standardized skill assessment, automated operative note generation, and the development of enhanced educational tools. This type of work in neurosurgery has been primarily limited to endoscopic procedures (2, 169). Khan et al. (2) conducted a study using ML to develop and validate an automated workflow analysis model for endoscopic transsphenoidal pituitary surgery, achieving high accuracy in recognizing surgical phases and steps (Figure 3). Pangal et al. analyzed the phases of endonasal endoscopic surgery using a validated cadaveric simulator of internal carotid artery injury (169).

In neurosurgery, CV has been increasingly applied to annotate or segment instruments and anatomical landmarks within operative videos (7). For instance, Jawed et al. developed a video annotation methodology for microdiscectomy, creating a standardized workflow to facilitate the annotation of surgical videos (168). Their method involved labeling surgical tools, anatomy, and phases in microdiscectomy videos. Similarly, Staartjes et al. conducted a proof-of-concept study evaluating machine vision algorithms for identifying anatomic structures in endoscopic endonasal approaches using only the endoscopic camera (170). The DL algorithm, trained on videos from 23 patients, significantly improved nasal structure detection compared to a baseline model, which is established using the average positions of the training ground truth labels within a semi-quantitative 3-tiered system.

Our ongoing work analyzing operative videos of middle cerebral artery aneurysm surgery to categorize, discriminate, and label surgical maneuvers or events with an ML methodology, however, suggests that the accuracy outcome depends on the information load presented to the analytical system (unpublished data). We have found that highly detailed, unique annotation and labeling are less accurate in identifying surgical maneuvers and events than more general labeling, suggesting that ML analytics has limits. This finding is intuitive and reflects the unique nature of each individual surgical procedure; the specific pathology, structure, site, and surgical maneuvers are different for each middle cerebral artery aneurysm.

In another study, Pangal et al. evaluated video-based metrics to predict task success and blood loss during endonasal endoscopic surgery in a cadaveric simulator (169). They manually annotated videos of 73 surgeons’ trials, focusing on instruments and anatomical landmarks. The study found that these metrics, derived from expert analysis, predicted performance more accurately than training level or experience, with a regression model effectively predicting blood loss.

Furthermore, in a recent study by Park et al. (17), computer algorithms including structural similarity, mean squared error, and DL were used to analyze the distortion, color, sharpness, and depth of field of the images collected from an advanced hybrid operating exoscope-microscope platform to improve the quality of the images for neurosurgical procedures. Operating microscopes are becoming sophisticated imaging platforms, incorporating fluorescence imaging, robotics, exoscopic vision, and now various proprietary functions for the enhancement of images to better define and visualize anatomy. These functions themselves operate on the basis of DL algorithms to enhance image sharpness and color and provide the operator with visual environments that allow improvements in capturing the depth of the operative field. Furthermore, while improving image quality, exoscopes do not significantly distort the images (17). These systems can provide high-definition images and help improve the recognition of structures during surgery.

#### 4.2.2 Outcome prediction

Neurosurgery involves high-risk procedures, making the use of AI for predicting outcomes a potentially important tool for improving surgical planning, patient counseling, and postoperative care. Outcome prediction in neurosurgery has dramatically benefited from advances in DL. These models can predict functional recovery and overall quality of life by analyzing preoperative imaging and intraoperative data, aiding in surgical planning and patient expectation management (25). DL models, such as CNNs and RNNs, are particularly effective in analyzing large datasets that may not yield meaningful patterns through traditional statistical methods. These datasets can include imaging, clinical records, and operative notes, allowing for more accurate outcome predictions.

The adaptability of DL makes it a valuable tool for personalizing neurosurgical care and improving long-term outcomes. For example, Jumah et al. (200) explored surgical phase recognition using AI and DL to improve outcome prediction in neurosurgery. Surgical phase recognition analyzes visual and kinematic data from surgeries to identify different phases of a procedure in real time. This capability enhances decision-making by providing surgeons with critical insights and alerts during high-risk phases, thereby reducing complications and improving surgical precision. This innovative approach contributes significantly to outcome prediction in neurosurgery. Additionally, Danilov et al. (136) used RNNs to predict the duration of postoperative hospital stays based on unstructured operative reports. This model demonstrated the potential of using narrative medical texts for making meaningful predictions, further illustrating the usefulness of DL in neurosurgery.

Wang et al. (85) developed a DL model to predict short-term postoperative facial nerve function in patients with acoustic neuroma. The study integrated clinical and radiomic features from multisequence MRI scans to enhance prediction accuracy. The CNN model achieved an area under the curve of 0.89, demonstrating superior predictive performance compared to traditional models in which various subtypes of ML are used (e.g., Nomogram, Light Gradient Boosting Machine). This predictive capability aids in surgical decision-making and patient counseling, allowing surgeons to anticipate facial nerve functional outcomes and tailor surgical approaches to minimize nerve damage.

In the context of traumatic brain injury (TBI), DL models used to enhance TBI triage have shown promise in predicting outcomes at hospital discharge, especially in low-resource settings where decision-making support is needed (30). These models offer a significant advantage in patient care and resource allocation, highlighting the growing role of AI in neurosurgical practice.

Furthermore, several studies have also demonstrated the potential of DL in predicting adverse outcomes, such as postoperative complications or prolonged hospital stays. Biswas et al. (31) introduced the ANCHOR model, an artificial neural network designed to predict referral outcomes for patients with chronic subdural hematoma. Validated using data from 1713 patient referrals at a tertiary neurosurgical center, the ANCHOR model demonstrated high accuracy (92.3%), sensitivity (83.0%), and specificity (96.2%) in predicting which referrals would be accepted for neurosurgical intervention. By integrating clinical features such as patient age, hematoma size, and medical history, the model supports clinical decision-making and potentially reduces adverse events by ensuring appropriate surgical referrals.

Pangal et al. (11) explored the SOCALNet model to predict surgical outcomes during hemorrhage events in neurosurgery. This neural network analyzes surgical videos to estimate the likelihood of achieving hemostasis, outperforming expert surgeons in accuracy. This model is valuable for real-time decision support, enhancing patient safety and surgical outcomes.

Survival prediction in neurosurgery has also been enhanced by DL approaches that integrate multimodal data, including imaging and genomic information. By accurately predicting survival probabilities, these models support clinicians in making informed decisions regarding treatment strategies and patient counseling (83). Di Ieva et al. (93) applied a DL model to predict outcomes in brain tumor management, focusing on survival prediction. The integration of imaging and genomic data enhances the prediction of patient survival, enabling better surgical planning and prognosis.

DL models enhance decision-making, optimize resource allocation, and improve patient outcomes by leveraging complex data from imaging, clinical records, and surgical videos. As neurosurgery continues to embrace AI, integrating DL technologies will be crucial in personalizing treatment strategies, minimizing risks, and ultimately elevating the standard of care in this high-stakes discipline.

#### 4.2.3 Movement analysis and microsurgical skill analysis via hand and instrument tracking

The application of DL in hand motion and instrument tracking in neurosurgery is an innovative and transformative topic. DL is capable of tracking both relevant and redundant surgical motions in real time and serves as an educational and forecasting tool for training the next generation of neurosurgeons. On the other hand, DL algorithms have also been used to track patient motor movements during functional neurosurgery procedures. In a study by Baker et al. (54), a DL-based CV algorithm was used for markerless tracking to evaluate the motor behaviors of patients undergoing DBS surgery. Intraoperative kinematic data were extracted using the Phyton-based CV suite DeepLabCut from the surgical videos of 5 patients who underwent DBS electrode implantation surgery, with comparison to manual data acquisition. The automated DL-based model showed 80% accuracy. Furthermore, a support vector machine model was also used in this study to classify patient movements. Classification by a support vector machine had 85.7% accuracy, including for 2 common upper limb movements: 92.3% accuracy for arm chain pulls, and 76.2% accuracy for hand clenches. This study emphasized the application of DL-based algorithms in DBS surgery to accurately detect and classify the movements of the patients undergoing surgery. Although the accuracy of a support vector machine for a specific type of movement was found to be low, the results are promising for future studies (54).

Koskinen et al. (12) investigated the use of a CV model to properly train an algorithm to accurately detect the movement of microsurgical instruments correlated with eye tracking. A DL approach using YOLOv5-1 composed of Cross Stage Partial Network allowed analysis of 6 specific metrics of path length, velocity, acceleration, jerk, curvature, and the intertool tip distance in 4 categories of surgical movements of dissection (to use a microscissor for vessel dissection), enhancement of the visual scene (to move objects away from the visual field to expose the dissection area), exploration with the tools (to find a new dissection plane), and intervention (to clean the bleeding from the field of focus). This novel DL application for an intracranial vessel dissection task was a successful proof-of-concept that demonstrated surgical movement tracking without sensors attached to hands or digits (12).

DL is widely used as a tool for educators and academicians, and its role in neurosurgical education is also prevalent. In a DL-based analysis of the surgical performance of surgeons of varying degrees of expertise, Davids et al. (131) analyzed videos of 19 surgeons by using a CNN and evaluated the skill levels using the area under the curve and accuracy. Novice surgeons showed a significantly higher median dissector velocity and a more considerable intertool tip distance, both of which served as discernment points between the experts and the beginners.

Microvascular anastomosis is another procedure used in neurosurgery that is highly dependent on the precision and accuracy of the surgeon’s intraoperative moves. Sugiyama et al. (171) employed a similar approach to Davids et al. (131) in using a YOLOv2 training system to train with microvascular anastomosis videos of surgeons of various experience levels ranging from novice to expert. Although experts could complete the tasks faster than the novice, nondominant hand results were observable only in certain phases of the simulated practice surgeries. This study introduced a DL-based video analysis algorithm (171).

Xu et al. (119) also studied the skill levels of surgeons performing microsurgery, developing a novel sensorized surgical glove by applying a piezoresistive sensor on the thumb of the glove. Additionally, this study used force-based data, meaning that the interaction between the surgical tools served as the basis for analyzing surgeon skillfulness. Similar to the studies mentioned above, this study also encompassed surgeons of varying degrees of expertise and compared various DL models, including long short-term memory and gate recurrent unit for movement detection. The study asserted that force data can yield discrimination between experts and novices, which means this concept could be used as an educational tool (119).

In a recent study conducted by Gonzalez-Romo et al. (174), a novel hand-digit motion detector incorporating an open-source ML model (https://ai.google.dev/edge/mediapipe/solutions/vision/hand_landmarker; MediaPipe, Google, Inc.) was developed using the Python programming language (Python Software Foundation, https://www.python.org/) to track operators’ hands during a microvascular anastomosis simulation (201). The model tracked hand and digit motion with 21 hand landmarks without physical sensors attached to the operators’ hands. Hand motion during the microanastomosis simulation was recorded with a neurosurgical operating microscope and an external camera. Six expert neurosurgeons performed the simulation, with interesting elucidation and comparison of their technical commonalities and variances using time series analysis. The hand-tracking system employed in this study is a promising example of tracking motion during surgical procedures in a sensorless manner (174). On et al. (172) used the same DL-based sensorless motion detection system (MediaPipe) to track operators’ hand motions during a cadaveric mastoidectomy. The procedures were recorded using an external camera, and the video output was processed to assess surgical performance and provide feedback.

Research on DL technology applicable to various neurosurgical educational settings is continuously growing, with the development of various scalable open-source solutions aimed at the goal of molding the next generation of competent neurosurgeons. The paradigm shift in neurosurgery, whereby real-time piecewise intraoperative motion can be evaluated, may ultimately improve postsurgical patient outcomes and optimize neurosurgeons’ efficiency in carrying out preplanned tasks and improvising to overcome unexpected surgical situations (172–174).

#### 4.2.4 Diagnostic support

Due to various factors, fast and accurate diagnosis in neurosurgery can be challenging (202, 203). Thus, DL algorithms have been employed to provide diagnostic support. This support includes segmenting urgent intracranial pathologies on CT to help with fast decision-making, identifying and classifying intracranial tumors and spinal pathologies for accurate treatment planning, and enhancing neuronavigation systems to achieve better surgical outcomes.

### 4.3 Specialized applications

#### 4.3.1 Applications in intracranial hemorrhages

Intracranial hemorrhages (ICH) are among the most challenging pathologies for neurosurgeons, particularly in emergency settings (204–206). Selecting the optimal treatment modality for ICH can be controversial; however, timing is crucial for choosing the appropriate treatment. DL algorithms could be used to segment ICHs on CT, aiding in selecting the most effective surgical strategy.

In a study by Tong et al. (13), the authors developed a 3-dimensional (3D) U-Net embedded DL model to segment intraparenchymal and intraventricular hemorrhages on CT (13). This study aimed to improve understanding of the boundaries, volume, and centroid deviation of each type of hematoma. By achieving this, the authors hoped to aid clinicians in selecting the most accurate catheter puncture path for treatment (13).

**Figure 4:**
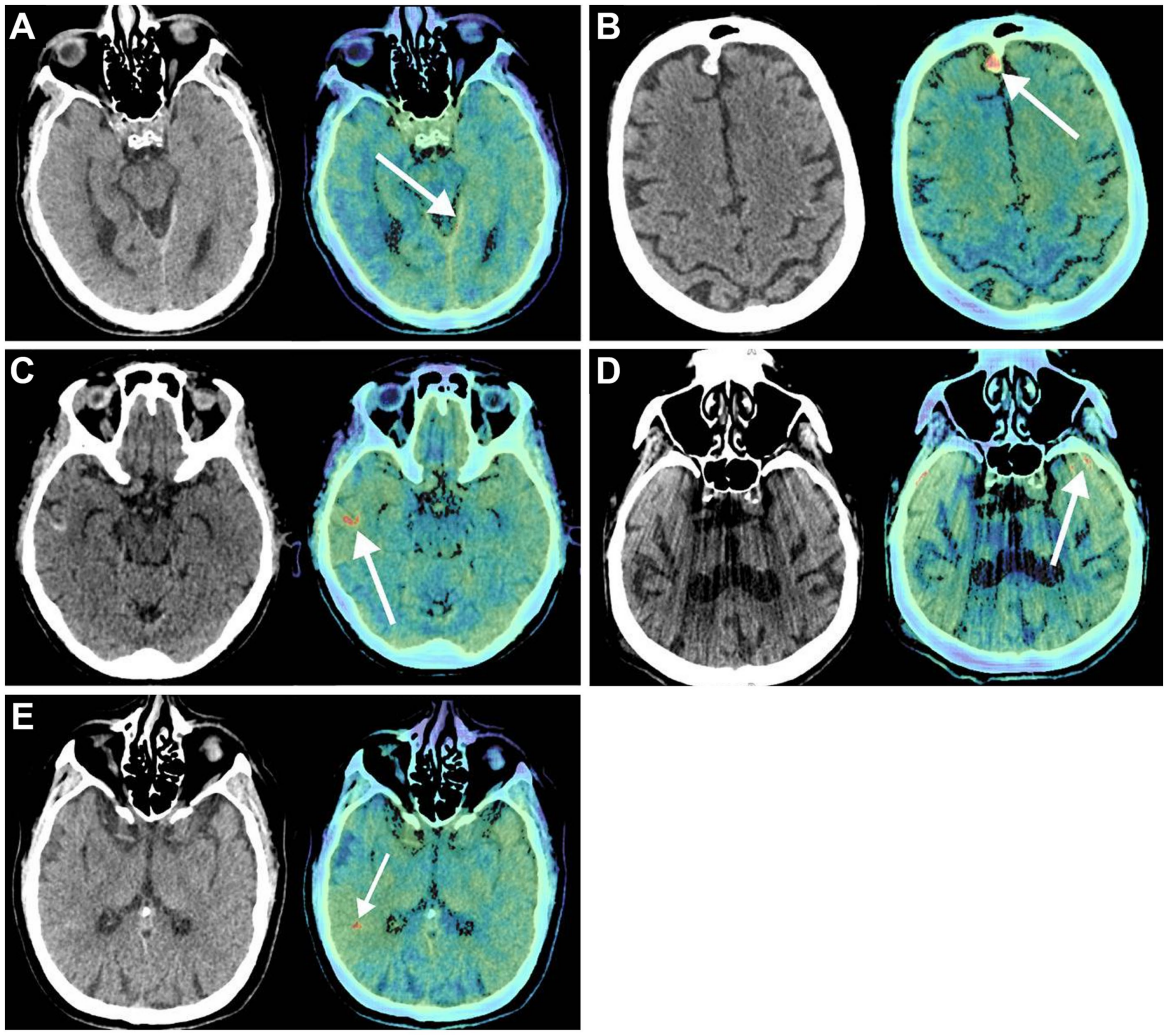
Deep learning (DL) in diagnostic support in neurosurgery. Voter et al. (38) focused on the application of a US Food and Drug Administration–approved DL, Aidoc, to determine its ability to recognize intracranial hemorrhages on noncontrast computed tomography (CT) accurately. Noncontrast CT (*left*) and the key images (*right*) in which the Aidoc identified the pathology (*white arrows*). **(A)** True positive finding in which the intracranial hemorrhage was not identified by the neuroradiologist. **(B)** Image of a meningioma that was incorrectly identified as an intracranial hemorrhage by Aidoc. **(C)** Cortical laminar necrosis incorrectly identified as an intracranial hemorrhage by Aidoc. **(D)** An artifact misidentified by the Aidoc. **(E)** Failure mode with the absence of a clear pathology. *Used with permission from Voter AF, Meram E, Garrett JW, Yu JJ. Diagnostic Accuracy and Failure Mode Analysis of a Deep Learning Algorithm for the Detection of Intracranial Hemorrhage. J Am Coll Radiol. 2021 Aug;18* (8)*:1143–1,152. Doi:10.1016/j.jacr.2021.03.005. Epub 2021 Apr 3. PMID: 33819478; PMCID: PMC8349782.*

Previously, the diagnostic accuracy of ICHs using DL models was tested in a retrospective study by Voter et al. (38). This study used a US Food and Drug Administration–approved DL model, Aidoc, to assess the diagnostic accuracy of ICHs using 3,605 noncontrast CT of adults (Figure 4). This study showed a decreased sensitivity and positive predictive value of the model compared to their expectations and previous studies, with specific patient features such as previous neurosurgery, hemorrhage type, and number of hemorrhages further reducing diagnostic accuracy. The authors raised concerns regarding the generalizability of these DL models. They additionally stressed the need to include patients with a prior history of a neurosurgical procedure when training these models and a more stringent standardization of study parameters in future studies (38).

The recognition of subarachnoid hemorrhages (SAHs) using DL has also been investigated. Nishi et al. recognized the difficulty in diagnosing patients with SAH (39). An AI system using a deep neural network architecture segmented noncontrast CT images from 757 patients with 3D U-net. Of these 757 patients, 419 had SAH confirmed by 2 neurosurgical specialists. Of these 419 cases, 392 were used to train the DL model, and 27 were used for validation. Image interpretation was conducted on 331 cases, which included 135 SAH cases and 196 non-SAH cases. The AI system demonstrated a high accuracy in diagnosing SAH, almost comparable to that of the neurosurgical specialists. Importantly, the system was useful in aiding in the diagnosis of SAH when used by physicians who were not specialists in neurosurgery, reflecting its potential use as a screening tool in settings such as the emergency room (39).

#### 4.3.2 Applications in neuro-oncology

Proper identification and classification of intracranial tumors are crucial for determining early management but might not always be successful on initial imaging (207, 208). Thus, auxiliary methods to improve this process are essential. DL algorithms have been widely employed in adult and pediatric neuro-oncology to accurately identify and classify intracranial tumors (175, 176, 209). These algorithms facilitate rapid diagnostic estimation and support the determination of the most appropriate treatment strategy (210). For this purpose and to thereby enhance their effectiveness, DL algorithms can be trained with MRI datasets to accurately predict tumor classification, or they can be applied to overcome the limitations of advanced real-time, cellular-scale imaging technologies, such as confocal laser endomicroscopy (CLE) and Raman scattering microscopy. As neurosurgery rapidly advances into an era in which such imaging technologies are increasingly employed intraoperatively, DL algorithms serve as a critical tool for improving diagnostic workflows, accelerating treatment selection, and ultimately optimizing patient outcomes.

##### 4.3.2.1 Confocal laser endomicroscopy

**Figure 5:**
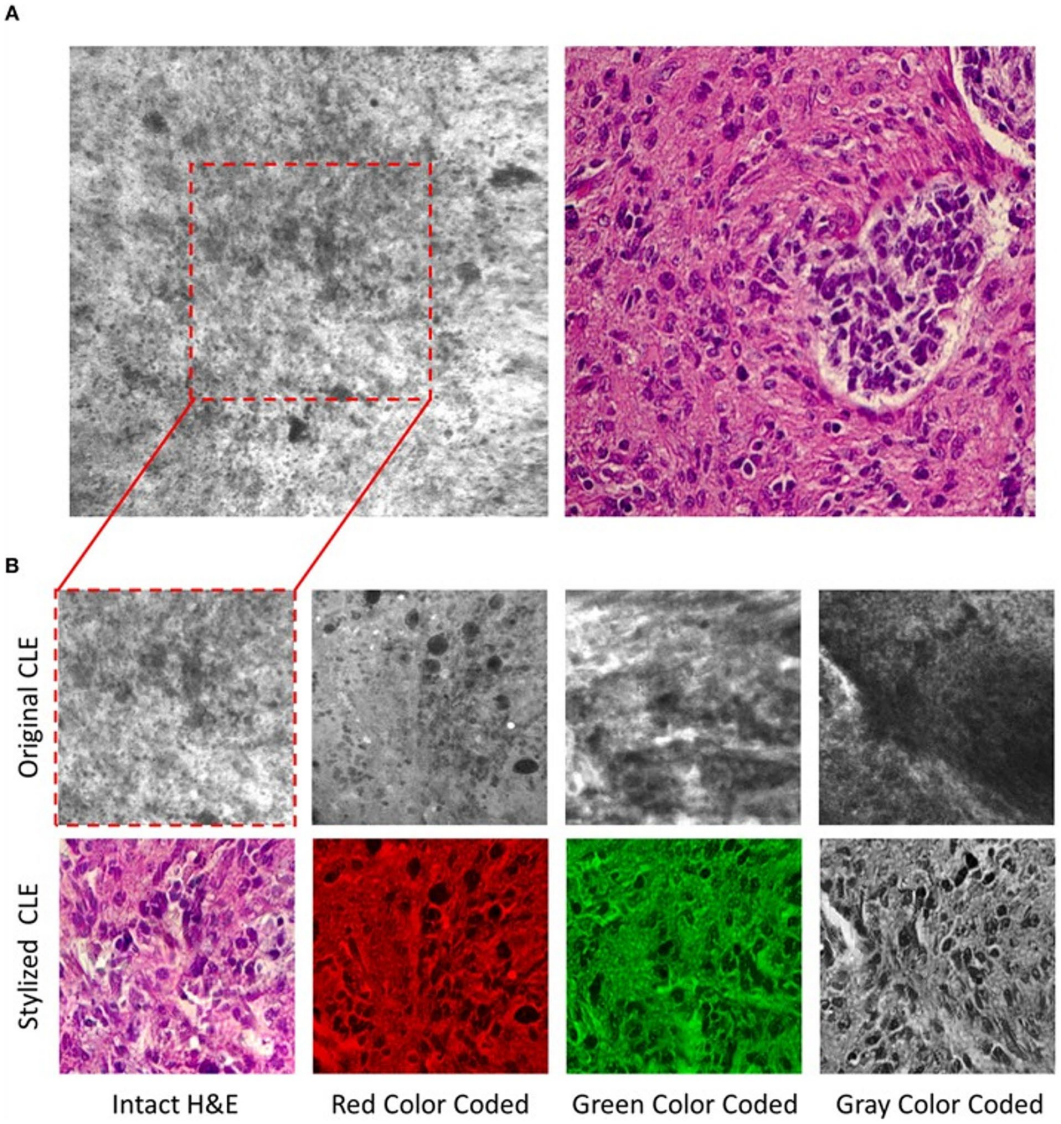
Deep learning (DL)–based image style transfer method to improve the diagnostic quality of confocal laser endomicroscopy (CLE) images. Image style transfer is a neural network–based model used in a study by Izadyyazdanabadi et al. (175) to integrate the content and style of 2 distinct images to transform fluorescence-based grayscale CLE images into familiar histologic hematoxylin and eosin (H&E)–like images. **(A)** Representative CLE (Optiscan 5.1, Optiscan Pty., Ltd.) and H&E images from human glioma tissues. The difference between original and stylized images of human gliomas can be seen on **(B)**, in 4 distinct color scales: intact H&E, red, green, and gray. *Used with permission from Izadyyazdanabadi M, Belykh E, Zhao X, Moreira LB, Gandhi S, Cavallo C, Eschbacher J, Nakaji P, Preul MC, Yang Y. Fluorescence Image Histology Pattern Transformation Using Image Style Transfer. Front Oncol. 2019 Jun 25;9:519. Doi: 10.3389/fonc.2019.00519. PMID: 31293966; PMCID: PMC6603166.*

CLE, a real-time *in vivo* intraoperative fluorescence-based cellular-resolution imaging technique used in brain tumor surgery, has the potential to revolutionize the surgical workflow in that it essentially provides a digital optical biopsy without requiring tissue extraction (176, 177, 209). Although promising, CLE devices are hand-held, extremely movement sensitive, and have a small field of view, making them prone to motion artifacts; they also provide images in grayscale only. To address the colorization limitation, Izadyyazdanabadi et al. (175) studied image style transfer, which is a neural network method used to integrate or rationalize the content of 2 distinct images in an attempt to transform the fluorescence-based grayscale CLE image into a familiar histology-standard hematoxylin and eosin–like image (Figure 5). Evaluation of the images by neurosurgeons and neuropathologists found that the transformed images had fewer artifacts and more prominent critical structures when compared to the original grayscale fluorescence-based images. This study emphasized an important application of DL technologies in neuro-oncology that enhances the diagnostic quality of intraoperative imaging techniques for better precision, which is particularly crucial for malignant and invasive brain tumors (175).

Because CLE imaging is sensitive to motion artifacts, it produces many images with nondiagnostic findings or limited surgical information (176). To address this discriminatory challenge, Izadyyazdanabadi et al. developed and used a DL method to detect diagnostic images among many nondiagnostic ones (176). AlexNet, a DL architecture, was trained with CLE image datasets collected from CLE-aided brain tumor surgeries, with all images verified by a pathologist. The mean accuracy of the model in detecting the diagnostic images was 91%; sensitivity and specificity were each also 91%. The results of this study showed that image detection and discrimination techniques based on a CNN have the potential to quickly and reliably identify informative or actionable CLE images. Incorporation of such techniques into the CLE operating system has the potential to aid the surgeon or pathologist in making an informed surgical decision on the fly when imaging with CLE.

Moreover, in another study (177), different CNN algorithms were trained using the CLE images of patients with different intracranial neoplasms to detect diagnostic images automatically. Accuracies of distinct CNN models were compared, and the study found that a combination of deep fine-tuning and creating an ensemble of models reached the maximum accuracy (0.788 for an arithmetic ensemble and 0.818 for a geometric ensemble). Using DL algorithms in intraoperative imaging techniques such as CLE has yielded promising results that may be a focus of future research.

##### 4.3.2.2 Raman scattering microscopy

Other intraoperative imaging has used DL image identification results, such as Raman scattering microscopy, which allows the generation of digitally stained histological images. Reinecke et al., using the residual CNN ResNet50v2, correctly identified tissue as tumor, nontumor, or low-quality tissue imaged with stimulated Raman histological images of intraoperative tissue samples (15). This study reported that the Raman histology-based residual network was 90.2% accurate in correctly classifying the different tissues compared to the classification conducted by neuropathologists, thereby aiding in surgical and clinical decision-making (15).

##### 4.3.2.3 Predicting tumor classification using MRI

Danilov et al. focused on predicting tumor classification by using contrast-enhanced T1 axial MRI images of World Health Organization (WHO)–grade verified glial tumors from 1,280 patients to train a DL model to accurately classify each tumor according to the WHO grading system (89). Two methods were used to achieve this goal: a 3D classification method in which the whole-brain MRI was used to predict tumor type and a 2-dimensional (2D) classification method using individual slices of each MRI scan. For model training, the processing of the 3D set was performed by the Dense-Net architecture, whereas the processing of the 2D model was performed by the Resnet200e architecture. The authors of this study concluded that the accuracy of their DL model in separating glial tumors based on the WHO grading system was similar to the results of other studies found in the literature (89).

##### 4.3.2.4 Use in pediatric neuro-oncology

Accurate classification of intracranial tumors is highly important because treatment strategies may vary accordingly, particularly in the pediatric age group. In this context, the use of DL for radiological diagnostic support has also been demonstrated in pediatric neuro-oncology to classify sellar and parasellar tumors accurately. In a study by Castiglioni et al., the ability of a DL model to determine the presence or absence of craniopharyngiomas on MRI of pediatric patients was assessed (80). To achieve this goal, the authors developed a CNN. They trained this model with sagittal MRI slices of the sellar-suprasellar regions of 3 groups: controls, craniopharyngiomas, and differentials (80).

The use of DL algorithms in neuro-oncology is crucial in enhancing early diagnostic capabilities for adult and pediatric patients. In this context, DL algorithms can be trained conventionally using MRI or employed to address the limitations of intraoperative methods such as CLE and Raman scattering microscopy, thereby enhancing their effectiveness.

#### 4.3.3 Applications in neuronavigation and neuroimaging modalities

##### 4.3.3.1 Neuronavigation

**Figure 6:**
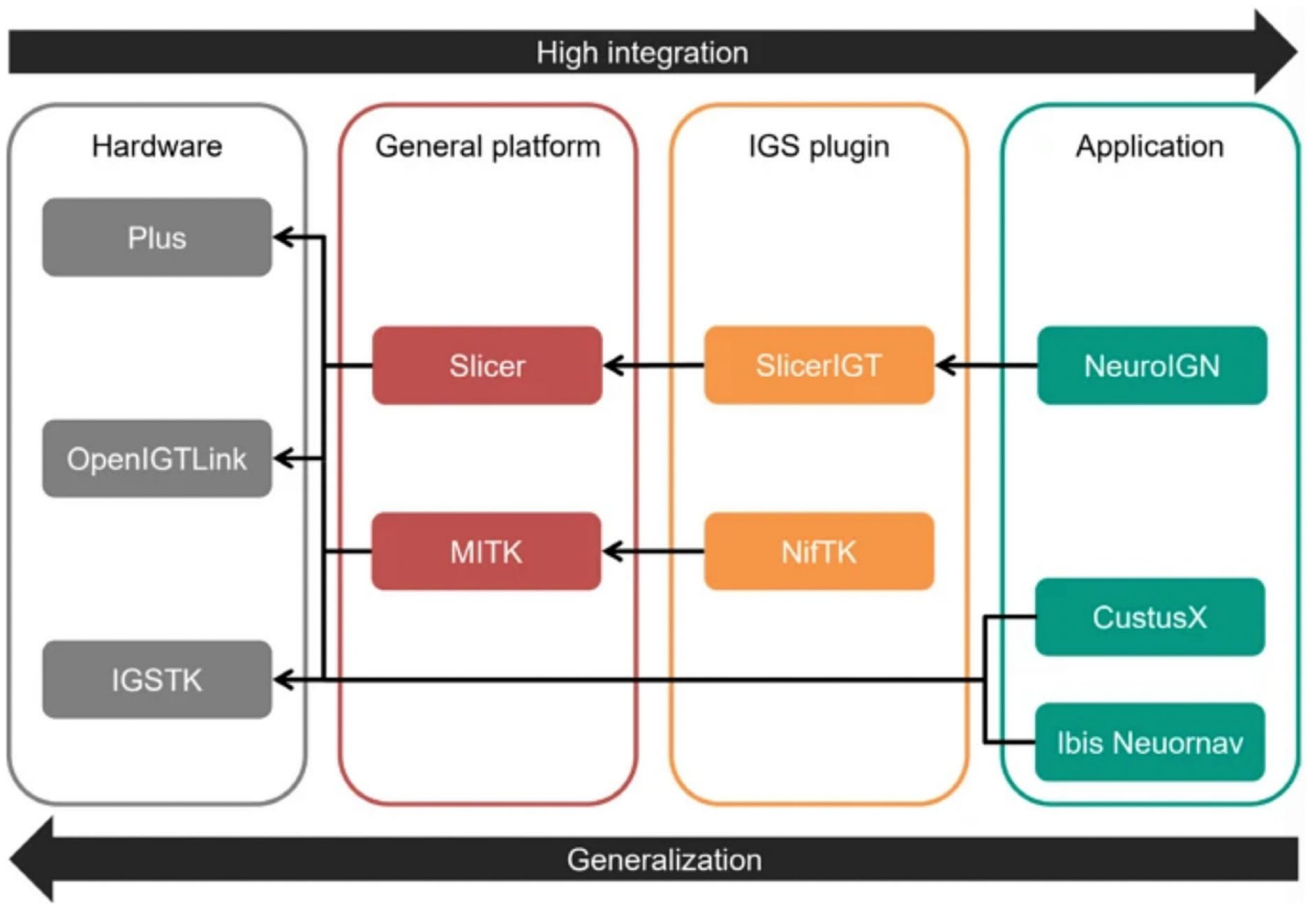
Role of deep learning (DL) in neuroimaging. The study by Zeineldin et al. (16) focused on developing a DL algorithm for tumor segmentation that includes explainable artificial intelligence techniques. This figure shows each level of the Neuro Image-Guided Neurosurgery (IGN) system, separated into hardware, general platform, image-guided surgery (IGS) plugin, and application. The hardware section is made up of 3 platforms: the Public software Library for UltraSound (Plus) platform, the Open Image-Guided Therapy Link (OpenIGTLink), and the Image-Guided Surgery Toolkit (IGSTK). The general platform level comprises the Slicer and Medical Imaging Interaction Toolkit (MITK). The IGS plugin level comprises the Slicer image-guided therapy (SlicerIGT) and the NiftyLink Toolkit (NifTK; NiftyLink). Finally, the application level includes the Neuro IGN system, CustusX, and Intraoperative Brain Imaging System Neuronavigation (Ibis Neuornav). The large arrow to the right shows increasing integration from left to right, and the large arrow to the left shows increasing generalization from right to left. The small arrows illustrate the dependency direction. *Used with permission from Zeineldin, R.A., Karar, M.E., Burgert, O. et al. NeuroIGN: Explainable Multimodal Image-Guided System for Precise Brain Tumor Surgery. J Med Syst 48, 25 (2024).* *https://doi.org/10.1007/s10916-024-02037-3*.

Neuronavigation systems aim to track surgical tools to ensure their real-time locations are precisely aligned with the patient’s anatomy. Various DL algorithms can also enhance these systems to increase accuracy and improve surgical outcomes. For example, NeuroIGN is a navigation system that integrates trained DL algorithms to recognize and segment brain tumors from MRI while including explainable AI techniques (Figure 6) (16). This study’s authors tested this system’s utility and accuracy to complete specific tasks such as registration and tracking, tumor segmentation, and real-time ultrasound imaging capabilities. They also evaluated how user-friendly this system was when used by individuals who had received only a short presentation on its use and were novices in using the NeuroIGN system (16). When evaluating the accuracy of the segmentation model, the authors observed that the system demonstrated good accuracy, making it an ideal candidate for image-guided neurosurgery (16).

**Figure 7:**
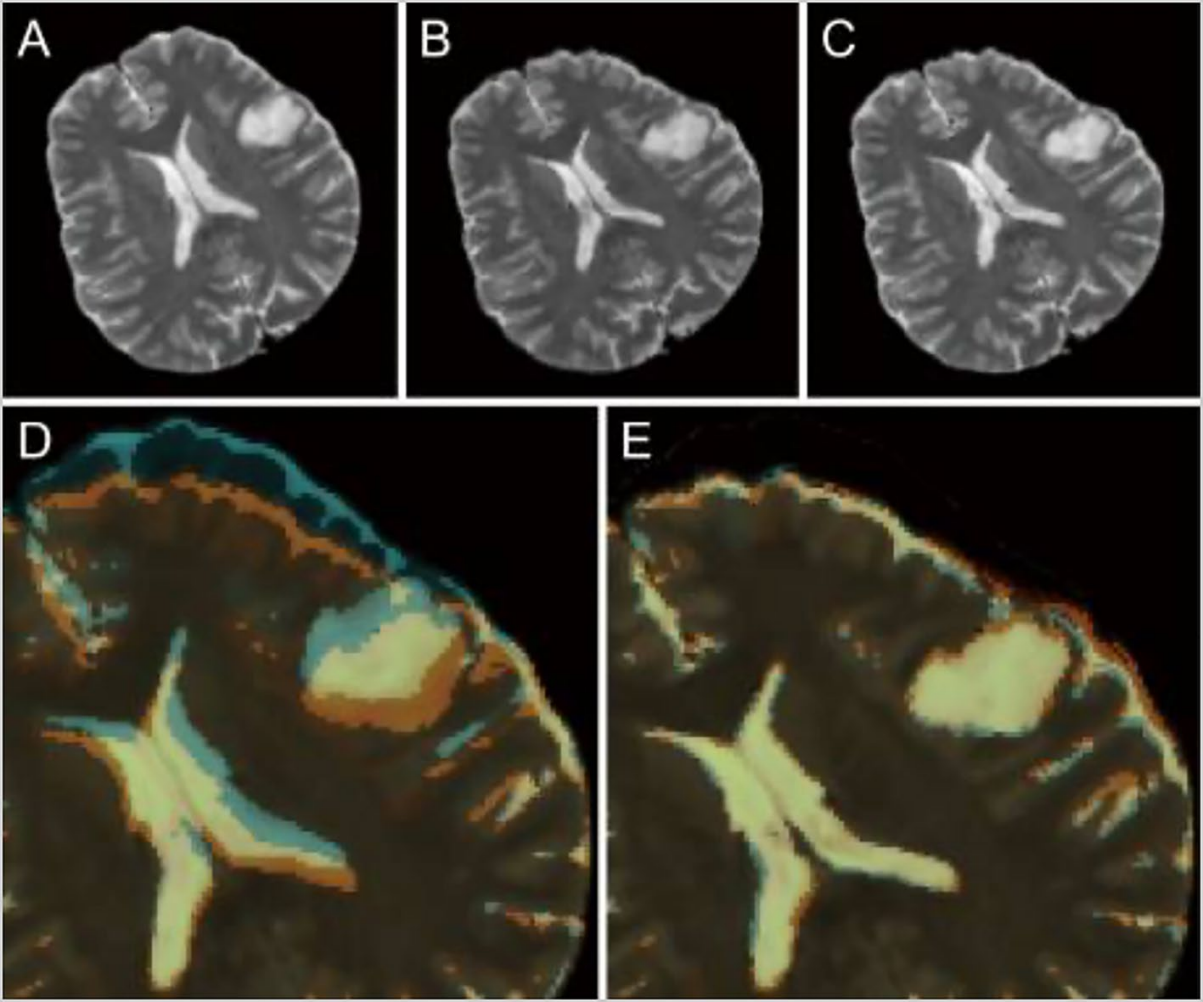
Role of deep learning (DL) in enhancing neuronavigation systems. A study by Shimamoto et al. (123) focused on the ability of a convolutional neural network (CNN) to adjust for and predict brain shifts based on preoperative and intraoperative magnetic resonance imaging (MRI). This figure illustrates the process of predicting brain shift intraoperatively compared to preoperatively in a selected case from the study (case 47). **(A)** Preoperative T2-weighted MRI. **(B)** Intraoperative image of the corresponding T2-weighted MRI. **(C)** The corresponding updated MRI. **(D)** The overlay of both the preoperative and intraoperative MRIs. **(E)** The updated MRI and the intraoperative MRI. The purpose of this image is to show the ability of the W-Net DL system to compensate for the brain shift. *Reproduced from Shimamoto et al., Neurol Med Chir (Tokyo), 2023. Licensed under CC BY-NC-ND 4.0.*

Neuronavigation during surgery is typically limited by brain shift following the incision and opening of the dura, which reduces the ability of neurosurgeons to identify their location intracranially during procedures. Shimamoto et al. used a CNN to design updated MRI to help remediate this issue (123). Preoperative and intraoperative MRI data from 248 patients was used, with the preoperative images serving as the training data for the CNN and the intraoperative images serving as the ground truth. This method allowed the model to learn how the brain shifted after the dural opening and adjust accordingly based only on preoperative images (Figure 7) (123).

Drakopoulos et al. aimed to tackle the issue of image distortion due to brain shift with the added obstacle of tissue resection by studying the utility of the Adaptive Physics-Based Non-Rigid Registration method (137). This study highlighted the limitations of neuronavigation systems that use rigid transformation and the need for a more accurate method to map patient coordinates once the brain has shifted. Preoperative images of 30 glioma patients were segmented into brain, tumor, and nonbrain regions, first by removing the skull and outer tissue with the Brain Extraction Tool (Oxford Center for Functional MRI of the Brain) and then segmenting with the 3D Slicer Software. The Adaptive Physics-Based Non-Rigid Registration method had superior accuracy in detecting tissue deformation compared to other systems for modeling deformation (137).

##### 4.3.3.2 Neuroimaging modalities

Zufiria et al. proposed a feature-based CNN to improve the accuracy of real-time interventional MRI during procedures such as electrode placements for DBS (129). This CNN was trained by simulating an interventional needle superimposed on 2,560 coronal and axial slices of T1 and T2 weighted MRIs from 1,200 patients. Using this feature-based reconstruction process to reconstruct interventional MRIs, this study aimed to increase real-time understanding of the exact locations of brain structures after brain shift in procedures such as electrode placements during DBS or biopsy procedures (129).

Zhang et al. focused on enhancing intraoperative cone-beam CT (CBCT) quality using a 3D DL reconstruction framework (153). The purpose of this study, similar to those mentioned above, was to improve the accuracy of neuroimaging following a brain shift. Although intraoperative CBCT is a cost and time-efficient imaging method, its drawbacks include a reduced soft-tissue contrast resolution, limiting its utility in intraoperative use. After training with simulated brain lesions from CBCT images, the 3D DL reconstruction framework’s efficacy was tested in neurosurgery patients to assess its reliability using clinical data (153).

DL methods have also been applied to improve the accurate identification and recognition of different shunt valves of cerebrospinal fluid shunts using radiographs (115). In a study by Rhomberg et al. (115), a CNN was trained using a dataset of 2,070 radiographs and CT scout images of shunt valves to recognize and correctly identify the model type. This study found that their CNN had a high accuracy in correctly identifying standard shunt models. The utility of this system stems from the necessity of recognizing different shunt models in patients with an unknown medical history (115).

**Figure 8:**
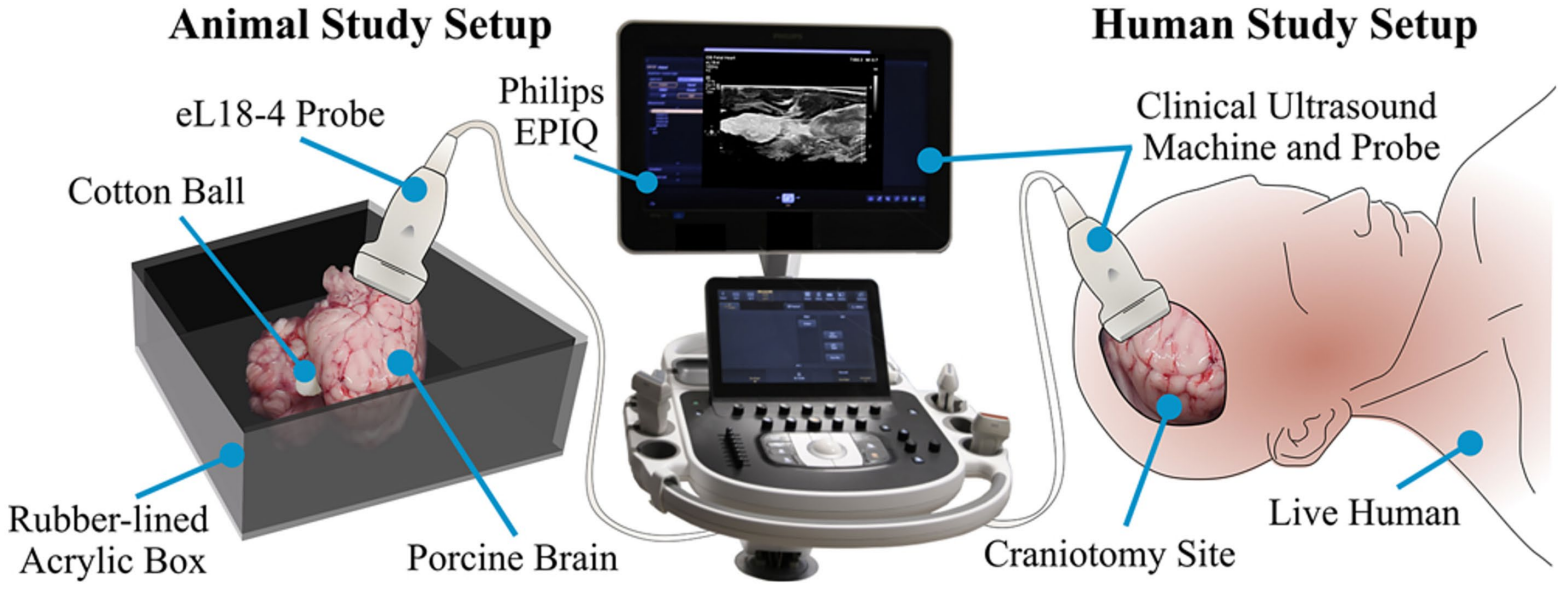
Deep learning (DL) in recognition of intracranial objects. The study by Abramson et al. (125) focused on developing an ultrasound-guided DL system that could ultimately recognize foreign objects left behind in patients’ surgical resection cavities. This figure illustrates the experimental design of this study. On the left, the authors started with testing the ability of the ultrasound DL system to recognize a cotton ball placed in porcine brains. When this test had been completed successfully, the researchers tested the ability of this DL system to recognize the presence of cotton balls within a human *in vivo* resection cavity. The ultrasound used in this case was the Philips EPIQ 7 using an eL 18–4 probe. Used with permission from Abramson et al. Automatic detection of foreign body objects in neurosurgery using a deep learning approach on intraoperative ultrasound images: from animal models to first in-human testing. *Copyright of Frontiers in Surgery and made available under the CC BY 2.0* (*http://creativecommons.org/licenses/by-nc-sa/2.0/*).

Recognition of objects intracranially using DL methods has also been applied to identify foreign bodies left behind following a neurosurgical procedure. Abramson et al. proposed an ultrasound-based system harnessing the powers of DL to help solve this issue (125). This study demonstrated the capabilities of their ultrasound system (using the Philips EPIQ 7 ultrasound machine) by first acquiring data by capturing images of a cotton ball implanted within porcine brains. In addition, the algorithm was tested to recognize a latex glove fragment measuring 5 mm in diameter, a stainless steel rod, and an Eppendorf tube. Following the success of these tests, cotton balls were placed within the resection cavities of 2 patients, with images captured by ultrasound used to train a DL detection algorithm (Figure 8). A custom version of the VGG16 CNN model demonstrated 99% accuracy in detecting foreign bodies within the brain (125).

The use of DL to identify specific abnormalities in neuroimaging modalities has also been examined in a study by Jiang et al. (28). This study analyzed noncontrast CT of patients with suspected TBI (28). This study focused on the ability of physicians with variable levels of expertise in neuroradiology or neurosurgery to detect a TBI compared with the ability of the DL model of icobrain TBI. Parameters used to detect TBIs included the presence of midline shift, hydrocephalus, hematomas, and Neuroimaging Radiological Interpretation System scores. The DL system’s ability to correctly categorize TBIs was similar to that of the attending physicians’ diagnoses. On the other hand, trainees had a lower level of agreement with the ground truth when compared to attending physicians. Moreover, they also demonstrated that, although the trainees had a substantial level of agreement on their initial review, using the DL algorithm as a supportive tool increased their agreement with the ground truth to an almost perfect level on their second review (28).

Although there are some limitations and controversies regarding their accuracy (27), DL algorithms can enhance diagnostic support. DL can facilitate surgical and clinical decision-making and aid in selecting the most appropriate treatment modality on a patient-specific basis, with particular auxiliary use in controversial cases and emergency settings.

##### 4.3.4 Volumetric assessment

Volumetric assessments are among the frequently used methods in neurosurgical studies for various purposes (211). However, since these assessments may require technical expertise, accurate measurements are not always achievable. Therefore, DL algorithms have increasingly been used to improve volumetric analyzes.

Measuring tumor burden is essential for evaluating treatment responses, particularly in neuro-oncology. Conventional 2D techniques are often unsuccessful in accurately measuring the volume of intracranial tumors, particularly gliomas, due to their irregular borders. Although still in development, DL models can address this challenge by providing more precise volumetric measurements for intracranial lesions.

An example of an intracranial lesion to which this method has been applied is pituitary adenoma (82). A study by Da Mutten et al. (82) created an automated volumetry pipeline to segment T1 contrast-enhanced MRIs of pituitary adenomas both preoperatively and postoperatively. This pipeline was developed by training a group of CNNs with 2D U-Net as the model architecture using manually segmented scans as training material. The model accurately segmented and completed a volumetric assessment preoperatively; however, the technique had difficulty achieving favorable results when assessing postoperative images. The authors hypothesized this may be partly due to interrater disagreement in ground truth segmentation of residual tumor tissue and image downsampling (82).

Tumor burden and volumetric assessment are often complex and limited due to the heterogenic and multifaceted nature of intracranial tumors. Chang et al. (18) studied automatic evaluation of the level of glioma burden employing the 3D U-Net architecture, specialized for detailed segmentation, by leveraging preoperative and postoperative MRI. A total of 239 patients were included in this study, which used automated processes to carry out tumor volumetric calculations. The study acknowledged that brain extraction, a step to discerning nonbrain tissue, was a rate-limiting step that created room for error during tumor segmentation (18).

A study assessing the feasibility of the application of DL in the extent of resection volumetric assessment of brain tumors was conducted by Zanier et al. (86). Single-institutional pre- and postoperative MRIs were manually labeled and combined with the Brain Tumor Segmentation Challenge 2015 and 2021 data from 1,053 patients. Use of U-Net architecture allowed the DL system to achieve faster and more accurate estimation of intracranial tumor volume (86).

Kang et al. (178) leveraged 12 DL models to automate the MRI segmentation process and obtainment of meningioma volumetric data. U-Net and nnU-Net–based DL training followed the manual segmentation. Although smaller than the Zanier et al. (86) study, the Kang et al. (178) study contained 459 subjects’ MRIs. nnU-Net, known to be better at image segmentation than U-Net, superseded the meningioma segmentation performance of U-Net, and 2D nnU-Net was the best performer. This study foresees the clinical applicability of this technology to manage specific meningioma cases (178).

The essential nature of accurately measuring tumor volumes warrants further research on the intersection between DL and volumetric assessment to optimize patient neuro-oncological surgical outcomes.

### 4.4 Challenges, ethical considerations, and future directions

The contribution of DL algorithms to reducing human errors and accelerating diagnostic processes in clinical practice is undeniable. However, their integration into medical and surgical practices faces several limitations.

One significant technical limitation is the heterogeneity of image datasets used for training DL algorithms (210). For instance, when MRI datasets are used to train these algorithms, the similarity of MRI signal intensities is crucial. Variations in signal intensity caused by differences in scanners can hinder the creation of homogeneous datasets, which can affect the reliability of outputs. To address this challenge, efforts must focus on standardizing the datasets to ensure more reliable and reproducible outputs (212, 213).

Another critical challenge is methodology bias. If the training datasets for these algorithms come from only one or a few centers, the DL model might unintentionally favor the imaging protocols unique to those centers rather than focus on the tumor’s more critical pathological features (210, 214). This bias can result in the DL model failing to generalize when presented with data from other centers with different imaging protocols. Considering the heterogeneity of brain tumors, this bias is a significant limitation. To mitigate this issue, datasets used for training DL algorithms should be extensive and, if possible, sourced from multiple centers with different patient populations. Additionally, incorporating imaging sequences with varying protocols during model training can enhance the assessment and segmentation capabilities of these algorithms (210, 214).

Performance evaluation presents another challenge. In some studies, the performance of DL algorithms in image segmentation is evaluated against the ground truth. However, the ground truth is typically determined through manual segmentation by radiologists or neurosurgeons, introducing subjectivity that can cause fluctuations in model performance. To address this, multiple manual segmentations should be performed, and their averages should be used as a reference to reduce human error (188, 210).

On the other hand, using imaging datasets composed exclusively of high-quality images to train DL algorithms raises the issue of selection bias. Although training with high-quality images may improve performance during the training process, real-world clinical scenarios often involve suboptimal-quality images in clinical settings. For this reason, the datasets used to train these algorithms can indirectly affect the clinical applicability of the outputs. Therefore, careful attention must be paid to the selection of datasets, and the final purpose of the algorithm should be thoroughly evaluated (188, 210).

In summary, large datasets with diverse images should be used for model training to maximize the effectiveness of DL algorithms in clinical practice. Selection and methodology biases must be carefully considered and minimized to ensure reliable and generalizable outcomes, and DL algorithms should be trained on datasets that accurately represent the target patient population. It should be noted that there is no guarantee that using DL algorithms in clinical settings will yield more accurate or efficient data, reduce process costs, accelerate results, or decrease latency for result appearance. Implementing DL processes requires substantial computing power and infrastructure. Furthermore, the results and outputs of DL algorithms are entirely dependent on the quality of the informational inputs.

The increasing use of DL algorithms in medicine and surgery presents significant ethical challenges. Standardized guidelines are required to evaluate the scientific integrity and clinical applicability of studies using DL algorithms. Ensuring reliability and reproducibility is crucial for guiding future research. Collaborative teamwork among surgeons, data scientists, and ethicists can play a pivotal role in creating robust standards and addressing these challenges (210, 215).

One of the critical issues is the “black-box problem,” which refers to the difficulty in understanding the connection between the input data used by DL algorithms and the output they generate. This lack of interpretability stems from the complexity of the processes within DL systems, often comprising numerous hidden layers. Despite this complexity, understanding these processes is essential to building trust in and refining these models and advancing their clinical applications (210, 216).

Patient privacy and data protection are also critical concerns. Although these algorithms are trained using extensive datasets, it is crucial to ensure the security and privacy of the data as the dataset size increases. DL algorithms must be trained with deidentified datasets to prevent the possibility of linking imaging data to the individuals they belong to. Institutions must prioritize privacy by applying strict deidentification protocols and fostering trust with patients through transparent communication (210, 217).

Addressing these ethical considerations through standardized guidelines, interpretability-focused advancements, and privacy-focused practices is essential for the responsible integration of DL in clinical settings (210, 218, 219).

In light of the challenges associated with the application of DL algorithms in clinical and surgical practice, future research should focus on several key points. Consolidated models can be developed to integrate various AI algorithms across the pre-, intra-, and postoperative spectrum to improve reliability. Achieving this will require standardized guidelines and effective collaborative teamwork (218).

In addition, integrating DL as a broad and widely available resource for clinicians will likely require significant monetary and infrastructural investment. Training programs will need to be implemented to inform clinicians about the use of these technologies and their diagnostic and computational limitations.

One avenue of future research could be to investigate the quantitative advantages of using DL in surgical practice from a time-saving or cost-saving perspective (218). Furthermore, the computational errors that these technologies may have could lead to technical errors that would directly affect a patient’s well-being. Future research should be directed toward not only how these technical errors can be minimized but also developing safeguards to avoid complete reliance on these technologies if errors do arise.

Despite the challenges, integrating DL technologies into surgical practice has the potential to significantly improve the surgical workflow from both operative and diagnostic perspectives. The ability to intraoperatively monitor a patient’s condition for adverse events or precisely determine tumor borders via neuronavigation could be invaluable for clinicians. The technologies discussed in this paper provide a general outline of how DL can be used. As research into these applications progresses, it is crucial for clinicians and patients to understand how and when to use these technologies. Simultaneously, the development of standardized guidelines and privacy considerations should be prioritized as the technical capabilities of DL continue to evolve.

Employing DL and other AI algorithms in neurosurgical practice requires a collaborative team composed of neurosurgeons, data and computer scientists, and bioengineers. Expertise in these fields is necessary for using DL algorithms in neurosurgical studies. It is advantageous for future research on this topic that the fields of computer and data sciences are among the most rapidly evolving fields in the scientific community.

## 5 Conclusion

DL technologies can potentially enhance neurosurgical practice in various beneficial ways. These include improving the surgical workflow through real-time monitoring and detection of adverse events and pathophysiological conditions in a diagnostic fashion. Moreover, DL can also potentially aid in training novice neurosurgeons by learning from the techniques of experienced neurosurgeons.

Future studies should focus on developing mechanisms to improve the ease of use and access to these technologies within the neurosurgical workflow and training physicians to understand their benefits and current limitations. Furthermore, future research should be guided toward training DL models using more diverse and robust data so that the diagnostic applications of these technologies can be expanded further.

## Data Availability

The data supporting this article will be made available by the authors on reasonable request.
